# Life-History and Spatial Determinants of Somatic Growth Dynamics in Komodo Dragon Populations

**DOI:** 10.1371/journal.pone.0045398

**Published:** 2012-09-19

**Authors:** Rebecca J. Laver, Deni Purwandana, Achmad Ariefiandy, Jeri Imansyah, David Forsyth, Claudio Ciofi, Tim S. Jessop

**Affiliations:** 1 Department of Zoology, University of Melbourne, Melbourne, Victoria, Australia; 2 The Komodo Survival Program, Denpasar, Bali, Indonesia; 3 Arthur Rylah Institute for Environmental Research, Department of Sustainability and Environment, Melbourne, Victoria, Australia; 4 Department of Animal Biology and Genetics, University of Florence, Florence, Italy; 5 Department of Wildlife, Conservation and Science, Zoos Victoria, Melbourne, Victoria, Australia; California State University Fullerton, United States of America

## Abstract

Somatic growth patterns represent a major component of organismal fitness and may vary among sexes and populations due to genetic and environmental processes leading to profound differences in life-history and demography. This study considered the ontogenic, sex-specific and spatial dynamics of somatic growth patterns in ten populations of the world’s largest lizard the Komodo dragon (*Varanus komodoensis*). The growth of 400 individual Komodo dragons was measured in a capture-mark-recapture study at ten sites on four islands in eastern Indonesia, from 2002 to 2010. Generalized Additive Mixed Models (GAMMs) and information-theoretic methods were used to examine how growth rates varied with size, age and sex, and across and within islands in relation to site-specific prey availability, lizard population density and inbreeding coefficients. Growth trajectories differed significantly with size and between sexes, indicating different energy allocation tactics and overall costs associated with reproduction. This leads to disparities in maximum body sizes and longevity. Spatial variation in growth was strongly supported by a curvilinear density-dependent growth model with highest growth rates occurring at intermediate population densities. Sex-specific trade-offs in growth underpin key differences in Komodo dragon life-history including evidence for high costs of reproduction in females. Further, inverse density-dependent growth may have profound effects on individual and population level processes that influence the demography of this species.

## Introduction

Somatic growth patterns represent a major component of an organism’s life-history [1], [2], [3]. Age-specific body size and growth rates represent complex organismal trade-offs reflecting energy allocation partitioned among growth, maintenance, storage and reproduction, so as to maximise fitness [4], [5], [6]. Variation in patterns of growth can result in fundamental variation among individuals and populations [6], and influence important life-history traits such as timing of sexual maturity [7], [8], [9]. Perhaps the most vital implication of growth is its effect on maximal body size, which influences competitive ability [10], survival [11], [12], and fecundity [13].

Sex-specific growth variation must occur to result in sexual size dimorphism (SSD), reflecting different tactics and requirements for energy acquisition and investment between maximum female and male body size [14], [15]. Female body size is typically shaped by fecundity selection [16], [17], [18], [19], whilst male size is driven largely by sexual selection [17], [18], [19], the forces for which may differ and can also depend on environmental variables. For size-dimorphic species in which males are larger, female growth rate must asymptote at a smaller size than males due to selection prioritizing reproductive investment at the expense of further growth [14].

Across a species distribution growth rates influencing life history and demography can vary considerably due to an interaction between genetic and environmental regulation [1], [20]. Studies have suggested a strong correlation between intraspecific growth variation and differences in resource availability [21], [22], [23]. Yet spatial variation in growth results from more complex interplay among resource availability and the efficiency with which an organism can assimilate energy [24], [25]; affected by inter- and intra-specific competition [26], [27]. On islands, inbreeding depression, a legacy of small and historically isolated populations could also influence somatic growth rates [20], [28].

Several issues have hindered understanding of the causes of individual growth rate variation in animals, and particularly ectotherms that experience indeterminant growth. First, most growth rate models do not accommodate potential polyphasic growth [29] and have ignored individual based autocorrelation in growth rate data inherent to longitudinal studies [30]. The recent development of non-parametric generalized additive models that include random effects (Generalized Additive Mixed Models; GAMMs) enable both polyphasic growth and individual effects to be accommodated in analyses [31]. Second, because long-term studies of marked individuals in multiple populations are required to evaluate the relative roles of genetic and environmental variables in determining growth rates, few studies have attempted to evaluate these factors in wild populations [32].

**Figure 1:**
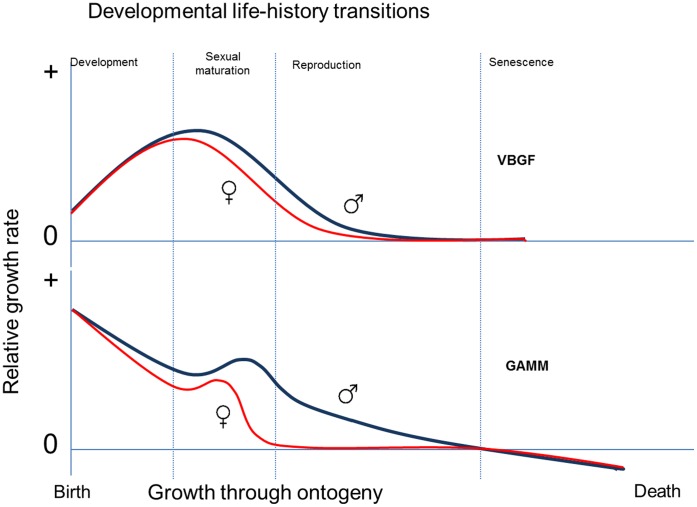
Comparison of varying models for growth trajectories in Komodo dragons. Contrasts the typical male and female trajectories expected for the von Bertalanffy growth function (VBGF; top panel) and generalized additive mixed model (GAMM; lower panel) for growth rate in relation to life-history transitions throughout ontogeny. Essentially because the GAMM is non-deterministic it was expected that maximum growth is predicted in the juvenile period of development whilst all surplus energy minus maintenance costs is channelled into growth. A dimorphic sexual maturation growth phase is predicted to occur with sex-specific differences reflecting different growth allocation decisions underpinning reproduction. Due to limited adult mortality associated with the dragons being apex predators, animals should be dying mostly as a result of old age, which potentially means there is a period of time when negative growth could be observed. These phenomena are not accounted for by the von Bertalanffy function.

Here, we used GAMMs and an information theoretic approach [33] to test hypotheses about the relative roles of genetic and environmental factors in determining somatic growth rates in ten populations of the world’s largest lizard, the Komodo dragon (*Varanus komodoensis*; 300 cm snout-vent-length and 87 kg body mass). Data were collected from marked individuals from 2002–2010, with the aim to evaluate individual, sex-specific and spatial patterns in somatic growth. It was assumed that several energy allocation trade-offs may alter investment in growth in Komodo dragons during life-history changes including distinct transitions in habitat use, sex related growth differences pre- and post-maturation, and aging, that may occur in long-lived apex predators such as this, which have very low mortality due to predation (Jessop, unpublished data). Each of these phenomena could result in polyphasic growth trajectories involving growth “lags” and “spurts” across ontogeny. As this sort of pattern may not be possible to model with the use of standard growth models such as the von Bertalanffy growth function (See Figure 1), the more flexible GAMM approach was used instead.

Next, sex-specific growth variation arising from the obvious sexual size dimorphism (SSD) in this species (∼70 kg in males vs. ∼25 kg in females) was addressed. Such pronounced SSD must entail different tactics and requirements for energy acquisition and investment [14], [15]. Again, GAMM comparisons were conducted to evaluate potential differences in growth patterns between males and females. To better evaluate if indeed differences in growth patterns were associated with sex-specific life-history differences we compared sex-specific size and predicted age distributions. If, as predicted, female Komodo dragons suffer higher mortality associated with higher costs of reproduction than males, then they should be under represented or absent from older age classes compared to males. If this is not observed it likely suggests males are subjected to higher costs of reproduction potentially due to male-male aggression when competing for females [14], [15], [19], [34].

The final aim considered spatial variation; looking at differences in ecological, demographic and genetic factors on the islands that may account for differences in growth patterns among sites. Dragon body size varies dramatically between islands, with the presence of dwarf and giant forms in part suggesting spatial variation in growth. For Komodo dragons potential variation in growth could be mediated by differences in prey availability [21], [35], differences in population density [26], [36] and, given the presence of high inbreeding coefficients in some lizard populations [37], inbreeding depression [20] could also underpin spatial variation in somatic growth rates among island populations.

**Figure 2:**
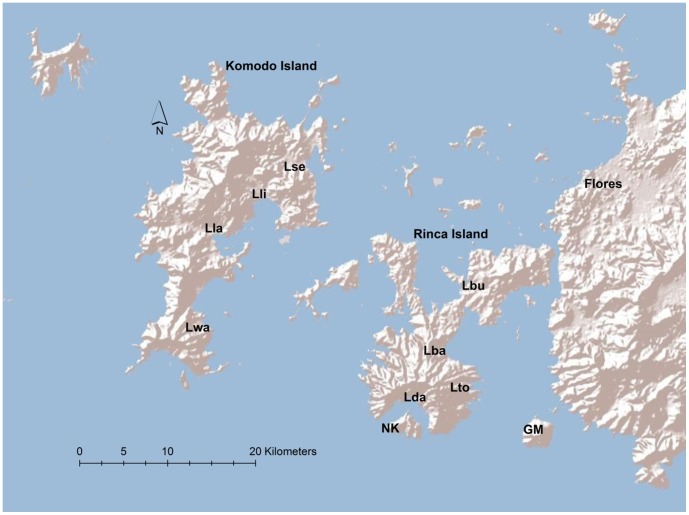
Map of Komodo National Park (KNP) and the location of the 10 field sites used in this study. Four sites were located on each of the large islands of Komodo (Lse, Lli, Lla, Lwa); and Rinca (Lbu, Lba, Lto, Lda). Single sites were located on each of the small islands of Gili Motang and Nusa Kode (Gm and Nk respectively).

## Materials and Methods

### Study Species and System

The Komodo dragon is endemic to five islands in eastern Indonesia, with four island populations in Komodo National Park and several fragmented populations on Flores [38]. The Komodo dragon is an apex predator, with three ungulate species dominating the diet of adults: rusa deer (*Rusa timorensis*), feral pig (*Sus scrofa*) and water buffalo (*Bubalus bubalis*) [35]. Previous work has shown the distributions and abundances of these three species to be important determinants of the demography of Komodo dragon populations [35].

To evaluate growth patterns in Komodo dragons, a capture-mark-recapture (CMR) study was undertaken at ten sites on four islands in Komodo National Park (8∶48:14.1 S; 119∶47:02.9 E), eastern Indonesia (Figure 2), during 2002–2010. The ten sites sampled (Figure 2) were: Loh Lawi (Lla), Loh Liang (Lli), Loh Sebita (Lse) and Loh Wau (Lwa) (all on 393 km^2^ Komodo Island); Loh Baru (Lba), Loh Buaya (Lbu), Loh Dasami (Lda) and Loh Tongker (Lto) (all on 278 km^2^ Rinca Island); and one site on each of the islands of Gili Motang (Gm; 10.3 km^2^) and Nusa Kode (Nk; 9.6 km^2^). Only two of 1062 marked Komodo dragons moved between any of the sites during the course of the study (T. S. Jessop et al., unpublished data) and hence the ten sites are considered discrete closed populations.

**Figure 3:**
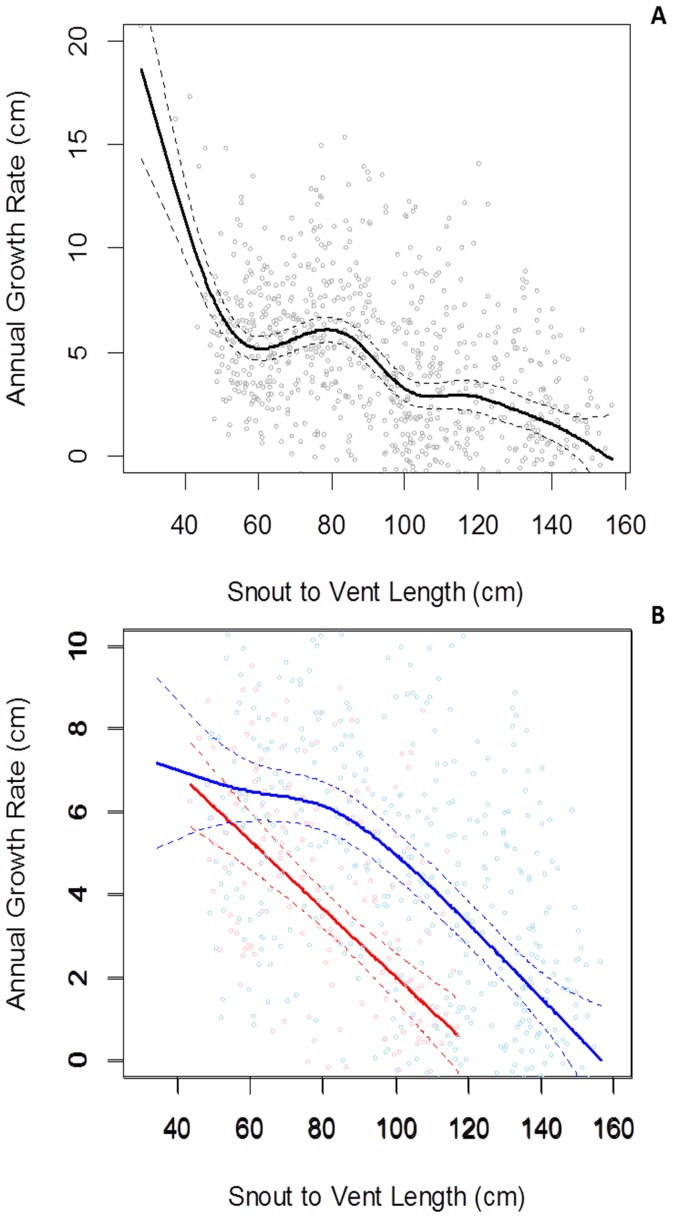
Size-specific growth curves (GAMMs; generalized additive mixed models) for Komodo dragons. These GAMMs model growth as predicted by mean body size (cm SVL; snout-vent-length). (a) All dragon growth records, (b) males (blue) and females (red/pink) compared. *Dotted curves* represent point-wise 95% confidence bands around fitted models. Note the female curve in (b) is not extrapolated beyond the maximum SVL at which animals were caught.

Dragons were captured in a trapping grid at each site using aluminium box traps and noose poles, and were uniquely marked with passive integrated transponder (PIT) tags (Trovan ID100; Microchips Australia, Melbourne). Further details of the capture program are outlined elsewhere [35], [36]. Snout-vent-length (SVL, cm), defined as the straight line distance measured between the tip of the snout and the cloaca to the nearest millimetre, was used as the measure of growth for each individual. The average of two measures of SVL, required to be within 0.5 cm of each other, was recorded to ensure increased precision of growth measurements. Measurements were made using a flexible fibreglass tailor’s tape. Sex was determined using molecular and morphological methods, outlined in detail in the supplementary material (see Methods S1). Briefly, molecular methods for sexing of lizards were conducted using genomic DNA extracted from blood samples. PCR amplification of sex specific alleles [39] was performed and patterns were then compared to those of a male and female whose sexes were previously verified from females conducting nesting activities and use of the largest individuals that represent males.

**Table 1:** Parametric and non-parametric terms of the generalized additive mixed model (GAMM) modelling growth rate of Komodo dragons.

Term		edf	*F*	p
**Non-parametric smooth**				
Mean body size (SVL) (cm)		7.462	41.696	**<0.001**
Year		5.645	8.422	**<0.001**
	Estimate	SE	*t*	p
**Parametric**				
Intercept	0.502	0.621	0.808	0.419
Site (Lba vs. Gm)	2.590	0.673	3.850	**<0.001**
Site (Lbu vs. Gm)	3.104	0.638	4.863	**<0.001**
Site (Lda vs. Gm)	1.432	0.701	2.044	**0.041**
Site (Lla vs. Gm)	1.327	0.667	1.988	**0047**
Site (Lli vs. Gm)	2.318	0.658	3.524	**<0.001**
Site (Lse vs. Gm)	1.966	0.706	2.786	**0.005**
Site (Lto vs. Gm)	1.814	0.677	2.678	**0.008**
Site (Lwa vs. Gm)	1.602	0.770	2.081	**0.038**
Site (Nk vs. Gm)	−3.572	1.415	−2.525	**0.012**
Sex (male vs. female)	2.433	0.315	7.734	**<0.001**
Sex (undetermined vs. female)	1.405	0.358	3.924	**<0.001**
R-sq.(adj) = 0.363, Scale est. = 9.477, n = 839				

*Notes:* SE: standard error; edf: estimated degrees of freedom for smooth term (1 =  linear); SVL: snout-vent-length. Probabilities (p) are bold if significant.

(<0.05). Site codes match those in Figure 2.

### Growth Rate Estimation

Absolute growth rates were calculated from growth records for each individual sampled using snout-vent-length as the dependent variable, and included negative and zero growth rates because animals can shrink in size due to senescence or in response to extreme resource limitation [40]. To minimise the effects of measurement error on growth rate estimation only dragons with recapture intervals greater than six months were included in our analyses. Details of further assessment of the relative effects of all available covariates; namely: sex, site, year, mean size (snout-vent-length; cm SVL), and recapture interval (years); on growth rate can be found in the supplementary material (see Methods S2, Methods & Results; Figure S1).

### Ontogenetic Growth Patterns

A two-stage statistical modelling approach was used to model somatic growth [41]. First a robust non-parametric regression model was fitted to the absolute growth rate data to derive the expected size-specific growth rate function dependent on potential growth predictors [31]. This function was then numerically integrated using a difference equation and a fourth-order Runge-Kutta integration method (M. Y. Chaloupka, pers. comm.) to derive the expected size-at-age growth function, which was finally numerically differentiated to produce the age-specific growth rate function. Further details on this approach can be found in [41].

Size-specific growth rates were modelled as a function of snout-vent-length using a GAMM [42]. The GAMM enables potential growth spurts associated with life-history changes, and the effects of individual heterogeneity, to be accommodated [30]. Year of capture and recapture interval were also included in the model to account for potential annual variation in growth and varying sampling intervals, respectively. GAMMs comparing the size-specific growth rates of each sex were also produced.

The mgcv package [42] in program R [43] was used to fit the models to the data. We evaluated the contribution of each covariate to the GAMM using *t*- and *F*-ratio tests [42].

### Testing Life-history Implications of Sex-specific Growth Patterns

Size frequency distributions were constructed for all male and female lizards using the SVL obtained at the first capture event. Using sex-specific age-at-size equations we predicted the mean age for each individual at first capture. To evaluate if male and female size and predicted age distributions differed significantly we performed Kolmogorov-Smirnov tests in program R [43].

**Figure 4:**
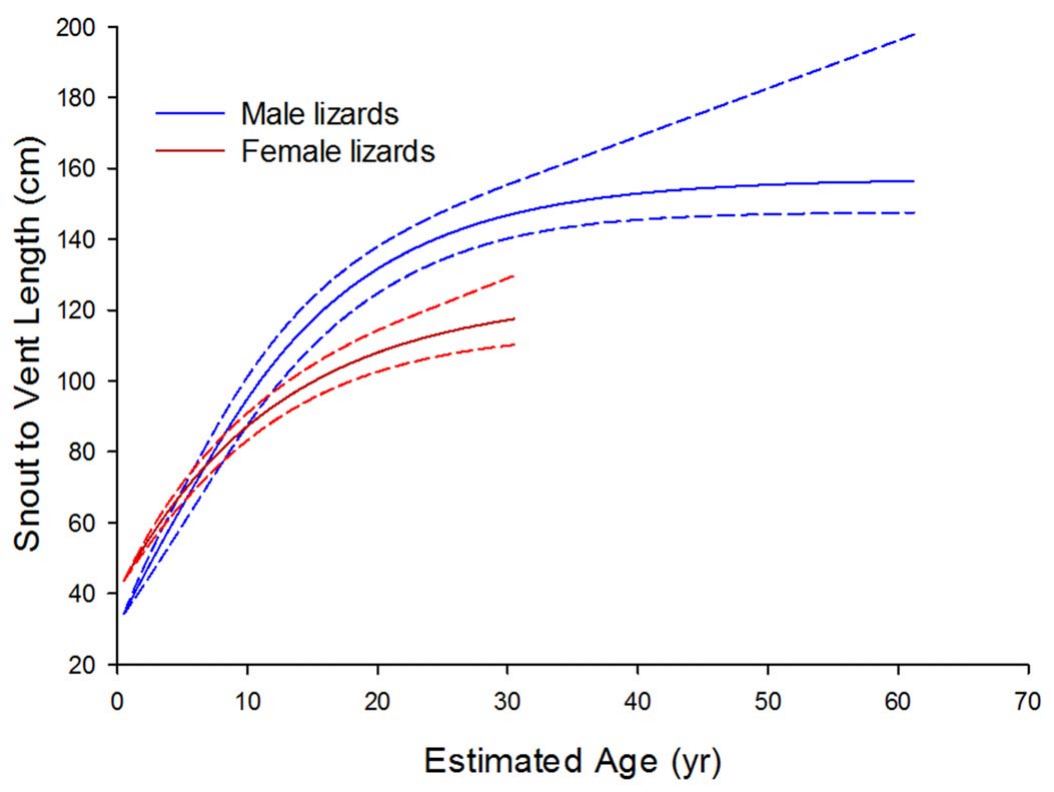
Size-at-age growth curves for Komodo dragons, derived by numerically integrating size-specific growth curves (**Figure 3B**). Male (blue) and female (red/pink) growth records compared. *Dotted curves* represent point-wise 95% confidence bands around fitted models. Note the female curve is not extrapolated beyond the maximum SVL at which animals were caught.

### Population Variation in Growth Rates

Four competing models were conceived involving three putative covariates; population density, prey availability, and level of inbreeding (site values can be found in Table S2); and an intercept only (null) model to explain spatial variation in somatic growth. Estimates of lizard density and ungulate prey availability for each site were calculated (means of 2002–2010 estimates), along with estimates of site-specific inbreeding coefficients (F*_is_*) (see methods below for determination of site estimates for each covariate). The four candidate models, including a null model, were fitted again using a GAMM approach in the mgcv package [42] in R [43], with dragon-specific heterogeneity and site as random effects. The relative support and ranking of the candidate models was assessed using Akaike’s information criterion (AICc) corrected for small sample size. The differences between each model’s AICc value and that of the best-fitting model were calculated (ΔAICc), with models of ΔAICc ≤2 considered to have substantial support, assuming that the (ΔAICc) for the null model was >2 [33]. Akaike weights (*w*), considering the strength of evidence that the candidate model is the best model for the data [33], were also calculated for each model.

**Figure 5:**
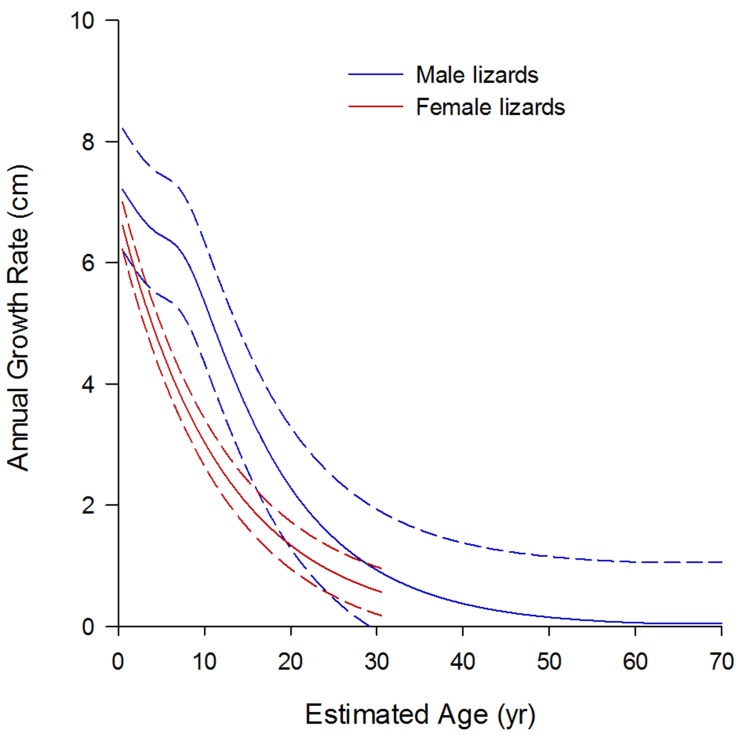
Age-specific growth curves for Komodo dragons, derived by numerically differentiating size-at-age growth curves (**Figure 4**). Male (blue) and female (red/pink) growth records compared. *Dotted curves* represent point-wise 95% confidence bands around fitted models. Note the female curve is not extrapolated beyond the maximum SVL at which animals were caught.

### Methods for Model Covariates

#### Population density

Jolly-Seber open population models in Program MARK (run in POPAN) [44] were used to estimate mean apparent abundance of Komodo dragons at each site between 2002–2010 (the period of contiguous animal trapping). Site density was then calculated by dividing the abundance estimate by the area of the trapping grid inflated to include a boundary layer [45] based on half the mean linear distance of individual movements recorded between annual recaptures within each site. This boundary layer accounts for the likely movement of lizards from outside the trapping grid.

#### Ungulate prey availability

Faecal counts were conducted annually from 2003–2010 (i.e. late dry season) at each site, with faeces of Timor deer and water buffalo counted within circular plots on 150 m transects in each site. Hand-held GPS (Global Positioning System, Garmin Summit, USA) units were used to locate transect line starting points, and ungulate faeces were counted within 30 sample plots placed at 5 m intervals along each transect. Between 20 and 41 transects were randomly positioned and orientated at each site, providing a total of 308 transects with a total length of 45.50 km. Faecal counts of both species show positive relationships with actual density estimates derived from distance sampling [46].

**Figure 6:**
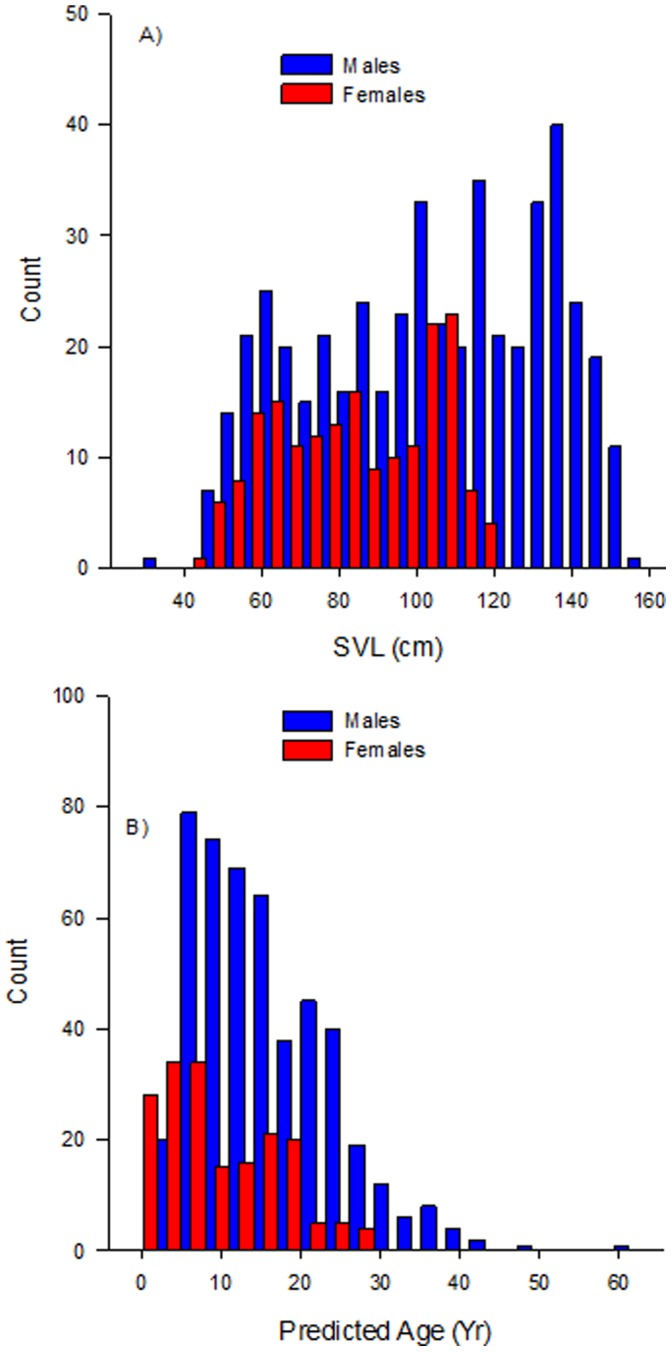
Sex-specific size and age frequency distributions for Komodo dragons. Comparison of male (blue) and female (red) frequency distributions in (a) body size (cm SVL) and (b) predicted age.

#### Inbreeding coefficients

Estimates of site-specific inbreeding coefficients were obtained predominantly from previously analysed *V. komodoensis* genetic microsatellite data sampled from eight sites [47], with additional microsatellite data collected from previously unsampled sites (Lla and Lba) collected during this study. In total 144 Komodo dragons were genotyped across 10 sites on Komodo (n  = 47), Rinca (n  = 56), Nusa Kode (n  = 9) and Gili Motang (n  = 12). Samples were screened for allelic variation at nine nuclear DNA microsatellite loci [48]. Polymerase chain reaction (PCR) amplification was performed using forward primers labelled with FAM, NED and HEX fluorescent dyes (Applied Biosystems). PCR conditions and thermal profiles were as described in [48]. The amplicons were resolved on an Applied Biosystems 3100 genetic analyser and allele sizes scored against a GeneScan 500 ROX size standard (Applied Biosystems) using GENEMAPPER 4.0. Estimates of site-specific inbreeding coefficients (F*_is_*) were calculated using Genetix 4.01 [49]. Statistical significance was obtained by comparing observed F*_is_* values to a frequency distribution of fixation indices obtained after 10,000 permutations of alleles.

**Figure 7:**
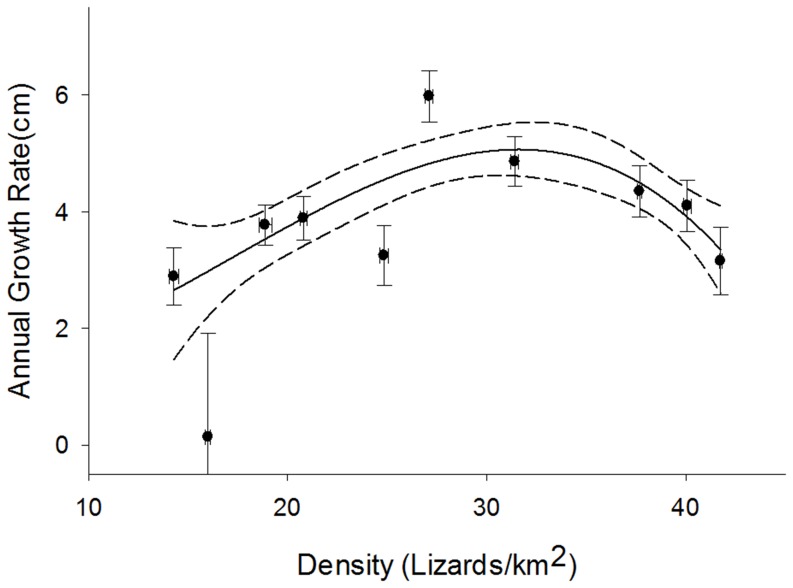
Relationship between population density (lizards/km^2^) at each site and site mean growth rates (cm SVL/yr). *Dotted curves* represent 95% confidence bands for the mean predicted curve. *Points* indicate mean growth rates for each site by population density. *Horizontal error bars* are standard errors of site population density means. *Vertical error bars* are standard errors of site mean growth rates.

### Research Permissions and Animal Ethics

This Research was authorized under successive collaborative research memorandums of understanding (MOU), first (2002–2007) between Zoological Society of San Diego, The Nature Conservancy and the Indonesian Department of Forest Protection and Nature Conservation (PHKA), and second (2008–2015) under MOUs between the Komodo Survival Program and PHKA. Animal experimental ethics committee approval was obtained from the University of Melbourne (under Permit 0911162.1).

**Table 2:** Model selection summary for the eight models explaining growth rate variation of Komodo dragons at ten sites in eastern Indonesia, 2002-2010.

Model	–ln(L)	df	AIC*_c_*	ΔAIC*_c_*	*w*
Lizard density	2308.83	6	4629.8	0.00	0.941
Inbreeding	2312.17	6	4636.4	6.69	0.033
Ungulate prey abundance	2310.93	8	4638.0	8.27	0.015
Null	2315.34	4	4638.7	8.96	0.011

*Notes:* The model in bold is the top ranking model. As well as providing the negative log-likelihood (–ln(L)) and AIC*_c_* for each model, df (degrees of freedom), ΔAIC*_c_* (difference between each model’s AIC*_c_* and the lowest AIC*_c_*) and the Akaike weight (*w*) are also shown.

## Results

### Scope of the Data

Growth measurements were obtained from 400 individually marked Komodo dragons (refer to Table S1) captured between 2002 and 2010. The data included records for 77 females, 201 males and 122 individuals of unknown sex predominantly spanning the post-arboreal phase from ∼28–157 cm snout-vent-length (SVL), with ≥54% of dragons recaptured during multiple annual sampling periods. The use of mixed-effects models incorporating individual ID as a random variable allowed for repeated measures so all recapture events greater than six months were used in the analysis. Recapture intervals used in this study ranged from six months to seven years, with a median of one year.

### Ontogenetic Growth Patterns

The estimated size-specific growth function for Komodo dragons was polyphasic with highest growth rate at the juvenile stage (Figure 3A), and included a growth spurt evident at ∼60 cm SVL, followed by another smaller spurt around ∼105 cm SVL. Growth begins a gradual decline after ∼120 cm SVL, reaching a point of zero growth at ∼158 cm SVL.

Comparing the estimated size-specific growth curves of both sexes (Figure 3B) however, shows an absence of growth spurts, with less complex growth patterns than the initial curve suggests. The female growth pattern is significantly different to that of the males (Table 1; Figure 3B) with females exhibiting a negative linear relationship between growth rate and size (cm SVL). Males in comparison showed a slow linear decline in growth rate followed by a faster linear decline with a distinct change at ∼60 cm SVL. Growth rates between sexes appear to begin to diverge relatively early, at least after a size of ∼42 cm SVL, though too few small individuals were sampled to provide a more accurate estimate.

### Age Dependent Growth Rates

The estimated size-at-age growth curves indicate that males and females are of similar size until ∼7 years (Figure 4), after which the growth trajectories diverge, with females tending towards a smaller maximum size than males. Females are no longer captured at sizes >117 cm SVL, whereas males grow larger, reaching an asymptotic size at ∼157 cm SVL at ∼62 years, also corresponding with the point at which growth rate asymptotes just above zero (Figure 5). Females, in comparison, fail to reach an asymptotic size, and the oldest females captured (∼31 years) were still growing (Figure 5).

The estimated age-specific growth function (Figure 5) indicates a gradually decreasing decline in growth rate with age in females, with males showing a similar yet delayed decline. It appears there is a pause in growth rate decline in males between ∼4–7 years, after which the decline resumes at roughly the same rate as the previous female decline.

### Testing Life-history Implications of Sex-specific Growth Patterns

Male and female lizards exhibited significantly different frequency distributions in body size with males exhibiting substantially larger body sizes than females (D  = 0.45, P  =  <0.001; Figure 6A). Similarly males and females exhibited significantly different frequency distributions in predicted age with males reaching older ages than females (D  = 0.19, P  =  <0.001; Figure 6B).

### Population Variation in Growth Rates

Significant differences in mean growth rates were observed among the ten sites (Table 1). Three of the four sites located on Rinca, the second largest island, had the highest mean growth rates observed (Figure S2), with the greatest mean growth occurring at Loh Baru (Lba, 5.974±0.358). The site on the island of Gili Motang had a smaller growth rate (GM, 2.883±0.495) than the lowest site means of each of the larger islands (Lwa, 3.149±0.551; Lda, 3.884±0.327), and the site on the smallest island, Nusa Kode, had the lowest mean growth rate (NK, 0.132±2.341).

Of the four candidate models conceived to describe spatial variation in growth, the lizard density model had the lowest AICc and received substantial support (*w*  = 0.94; Table 2). The form of the relationship between population density and growth rate was concave down, with highest growth rates occurring at intermediate population densities (c. 32 dragons km^−2^; Figure 7). The other two candidate models (i.e. inbreeding, ungulate prey abundance) and the null model all received little support (Table 2).

## Discussion

Somatic growth dynamics of individuals and populations can have profound consequences for fitness, life-history and demography [1], [2], [3]. Here we demonstrate that growth dynamics of the Komodo dragon (*Varanus komodoensis*) exhibited: distinct sex-based differences in growth trajectories, suggesting altered energy allocation tactics with different life-stages; and spatial variability in growth rates, due to population density-dependent processes.

In species with indeterminate growth, where females have higher energy costs for reproduction, they must allocate nearly all energy investment from growth to reproduction after maturity (thought to be ∼8–11 years of age in females); whereas males may typically invest considerably less to ensure reproductive success increases with body size [50], [51], [52], [53]. Sexual selection instead drives males to continue heavy energy investment into prolonged growth past maturity as larger males gain greater advantage in agonistic encounters over females and territory [34], [54], [55]. These fundamental allocation differences between sexes hence necessitate different growth strategies. The different growth trajectories of male and female Komodo dragons (Figure 3B) provide evidence for different energy allocation strategies between the sexes. Females display a faster, linear decrease in growth rate compared to a delayed and slower decline in growth rate in males. This results in females being smaller than males. Moreover, slower female growth results in a smaller body size, without an asymptote in growth rate, possibly inferring the particularly high costs of reproduction and ensuing high survival costs [19], [56].

The largest females tend to be in poor body condition (Jessop, unpublished data) due to extended periods of fasting whilst nest-guarding. Males in comparison appear to live longer presumably aided by lower reproductive costs and an absence of predation conferring higher male survival. A consequence of these sex related differences appears to be large differences in the age of maturity and also maximum longevity estimates (Figure 4, Figure 6). Female maturity is estimated here at around 8–11 years based on the smallest body size of known nesting females. More importantly female longevity appears to be considerably truncated at ∼31 years compared to males, after which females are no longer captured signalling their absence within the population beyond this age (Figure 6B). Asymptotic size for males however, is estimated at ∼62 years and presumably they can live considerably longer than this during post asymptotic growth phases. Like many large reptiles, Komodo dragons are long-lived, which has broad implications for their population dynamics [41]. In particular, this vast difference of ∼30 years in longevity between sexes (Figure 6B) could have dramatic consequences for population demographics in terms of male biased sex-ratios. Not only would this require consideration in planning any conservation efforts for populations on the brink of extinction [57], [58], but skewed population sex ratios due to precocious female deaths could be exacerbating competition between males over remaining females, which would in turn increase sexual selection on males to grow even larger [54], [55].

Significant variation in mean growth rates was evident among sites and islands (Table 1, Figure S2). Support for lizard density-dependent effects on spatial variation in dragon growth rate was substantially higher than alternative models. Prey availability and inbreeding had no detectable influence upon growth rate (Table 2, Figure 7). Implicit with density-dependence is elevated intraspecific competition [59], [60] and agonistic social interactions [24], [61], [62] that reduce an individual’s foraging ability [27]. For instance, following optimal foraging theory it could be assumed that intraspecific competition for resources may influence the diets of less successful competitors resulting in reduced opportunity to select more profitable prey or perhaps even forcing alteration of foraging tactics [63], [64]. Density-dependent effects are influential determinants of individual and geographic variation in somatic growth dynamics [26], [27], [65]. The low growth rates of Komodo dragons observed at high population densities could arise due to effects of overcrowding increasing competition for resources, as well as increased energy expenditure on intraspecific interactions, including heightened agonistic encounters and resulting stress [66]. However, density-dependent effects on growth were curvilinear (Figure 7) with low growth rates at low population densities. In other words, the additional inverse density-dependence at lower dragon densities suggests an Allee effect [67]. Allee effects arise due to genetic effects of inbreeding and loss of heterozygosity, demographic stochasticity such as fluctuating sex-ratios, and reduction in cooperative interactions between individuals important for successful mating encounters [67]. In Komodo dragons, inverse density dependence in growth rate is most significant on the two smallest islands where the lower growth rates could be a product of both the environmental and genetic processes [68]. The low density populations with low growth rates (Figure 7) are possibly of concern as this could be an indication of populations in decline and at risk of local extirpation [36].

With size-specific growth variation among sites and islands, it would be worthwhile to detect specific habitats with distinct environmental or genetic differences in dragon growth trajectories that underpin life-history traits such as maximum body size, longevity and vital rates. Vast geographic variation in phenotypic traits are recognised in various intra- and inter-specific studies of sea turtles [27], [69], [70], fish [71], lizards [72], and birds [73].

Our study shows that across ontogeny somatic growth of Komodo dragons is polyphasic in males, yet not in females, as a consequence of differences in energy allocation tactics regarding the onset of reproduction, which also result in an extreme contrast in longevity between sexes. This vast difference in life-span likely has a significant effect on population sex ratios, which in turn could be increasing sexual size dimorphism (SSD) within this species. In future it would be interesting to compare how SSD may vary between populations in regards to demographics and environmental quality [55]. The density-dependent effects on growth rate observed in this study likely signal negative demographic consequences for certain populations as a broader consequence of poor environmental conditions. As a unique and endangered species with an already limited range, better understanding of how sex-specific and population differences may affect growth rates with resulting consequences for population demography, may be extremely important for future conservation of Komodo dragons.

## Supporting Information

Figure S1 **Mean growth year index as a predictor for growth rate in Komodo dragons.** Year index was one covariate in the fitted generalized additive mixed model (GAMM). *Dotted lines* represent the 95% confidence interval for the fitted values.
(TIF)

Figure S2 **Spatial variation in growth rates in Komodo dragons.** Mean growth rates (cm SVL/yr) for each site indicate spatial variation in growth among sites and islands. *Error bars* are standard errors of site mean growth rates.
(TIF)

Methods S1 (DOCX)

Methods S2 **Methods & Results.**
(DOCX)

Table S1 (DOCX)

Table S2 (DOCX)
