# Supplementary figures and images for: Life-History and Spatial Determinants of Somatic Growth Dynamics in Komodo Dragon Populations
Source: PLoS One. 2012 Sep 19;7(9):e45398. doi: 10.1371/journal.pone.0045398 (PMC3446886; doi:10.1371/journal.pone.0045398)

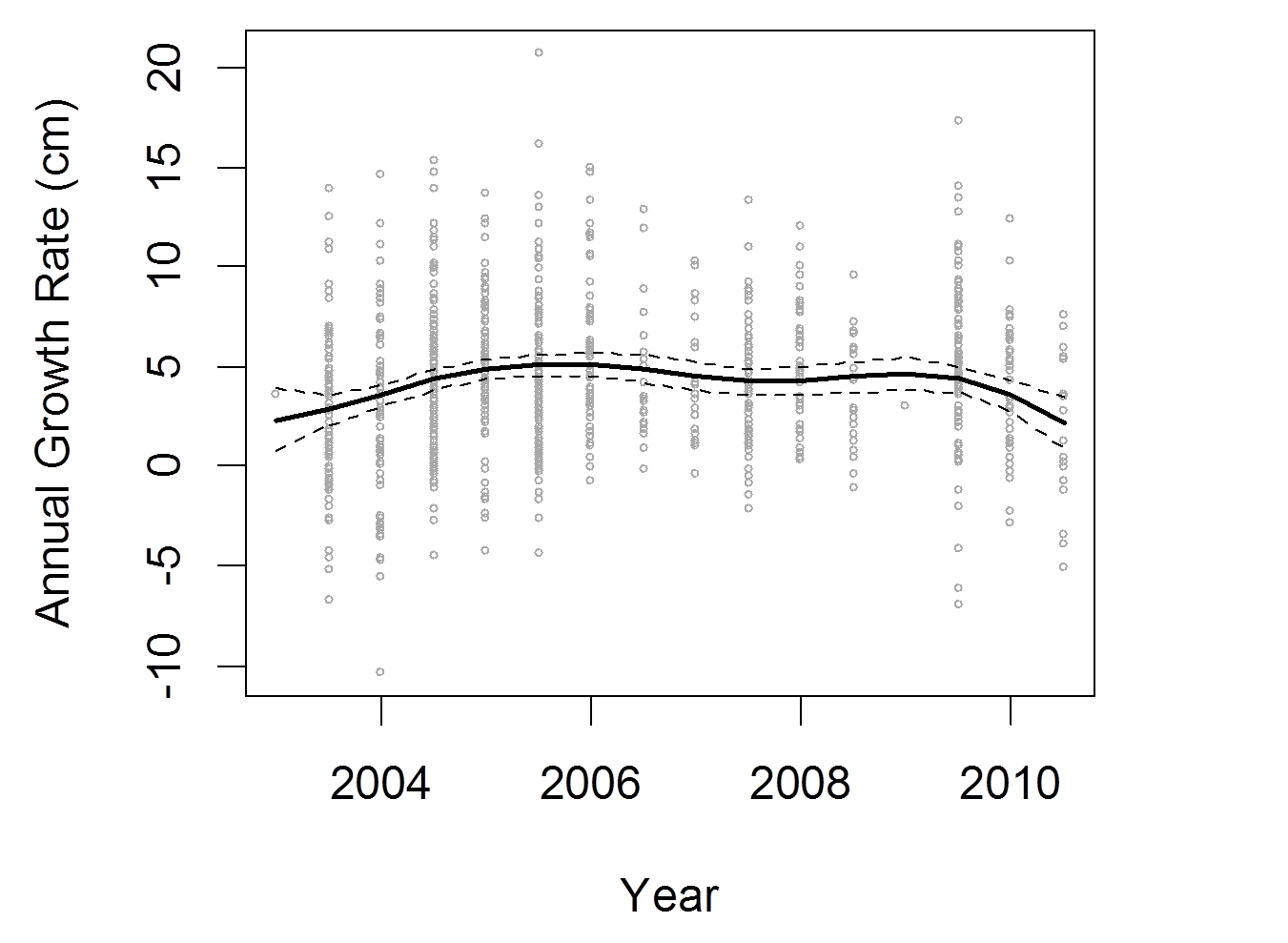

Supplement: Figure S1 — Mean growth year index as a predictor for growth rate in Komodo dragons. Year index was one covariate in the fitted generalized additive mixed model (GAMM). Dotted lines represent the 95% confidence interval for the fitted values. (TIF) [file pone.0045398.s001.tif]

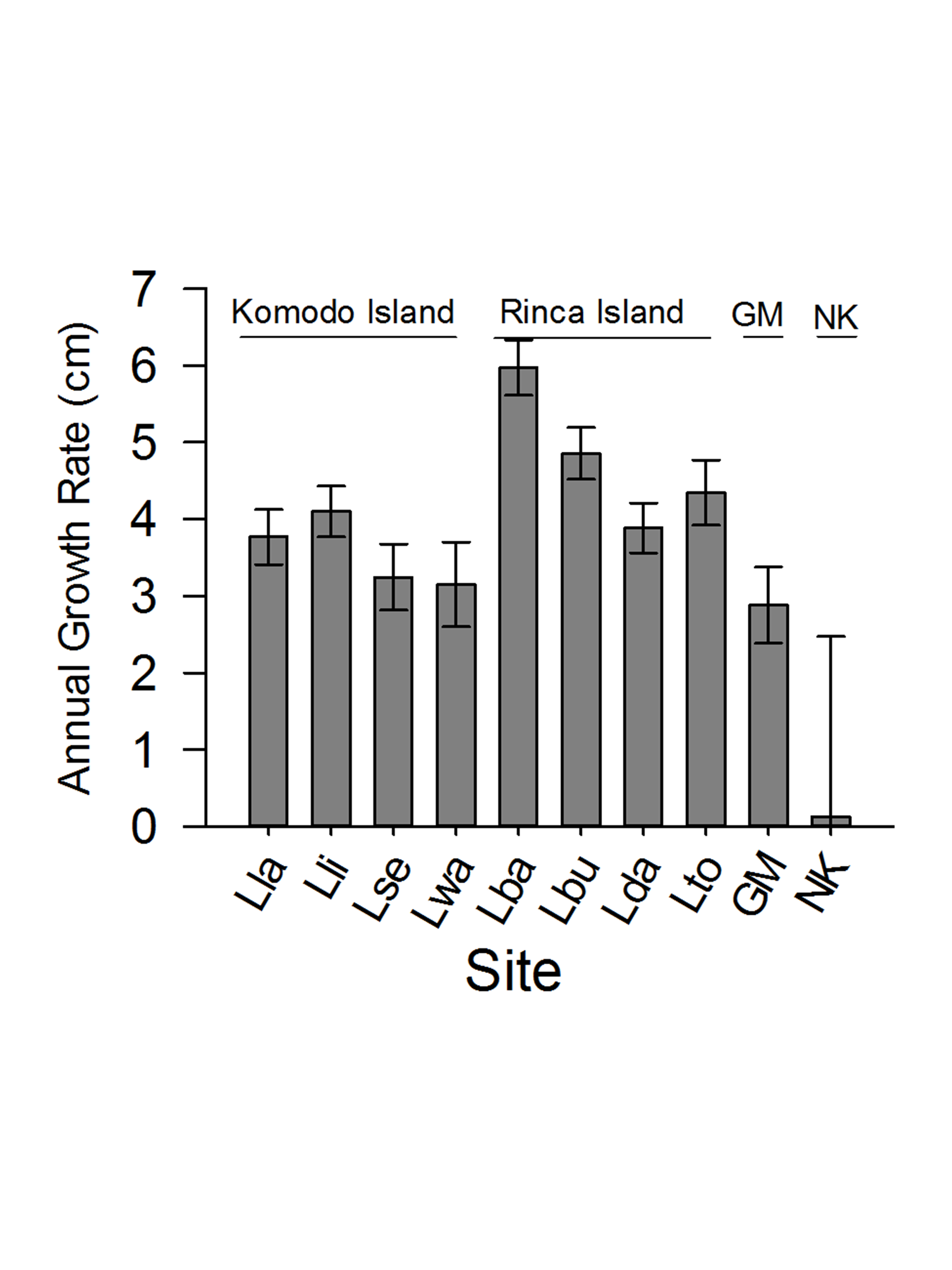

Supplement: Figure S2 — Spatial variation in growth rates in Komodo dragons. Mean growth rates (cm SVL/yr) for each site indicate spatial variation in growth among sites and islands. Error bars are standard errors of site mean growth rates. (TIF) [file pone.0045398.s002.tif]
