# Supplementary material for: Life-History and Spatial Determinants of Somatic Growth Dynamics in Komodo Dragon Populations
Source: PLoS One. 2012 Sep 19;7(9):e45398. doi: 10.1371/journal.pone.0045398 (PMC3446886; doi:10.1371/journal.pone.0045398)
Supplement: Methods S1 — (DOCX) [file pone.0045398.s003.docx]

**Supplementary Methods S1:**

*Sex determination*

Genomic DNA was extracted from blood in lysis buffer using a DNeasy Blood and Tissue Kit (Qiagen), following the manufacturer’s protocol. To determine potential sex ratio differences between treatments, lizard blood samples were genetically sexed using PCR primers that amplified sex specific alleles [39]. Amplifications were performed in a 20µL total volume, containing 2µL of DNA (diluted 1:10 in TE buffer), 10µL Gotaq (Promega), 0.5µL of each primer (10µM) and 7µL of H_2_O. PCR amplifications were performed on a Corbett Palm-Cycler using a touchdown thermal cycle program with the following parameters: initial denaturation at 94°C for 5 mins, followed by two cycles of 94°C for 30 sec, an annealing step at 65°C for 30 sec and 72°C for 90 sec; 2 cycles each with annealing temperatures of 60°C, 55°C and 50°C; 30 cycles with an annealing temperature of 48°C; then a final extension step of 10 mins at 72°C. Amplifications were checked on a 1.2% agarose gel and amplification patterns were compared to those of a male and female whose sex had been verified anatomically.
