# Supplementary material for: Life-History and Spatial Determinants of Somatic Growth Dynamics in Komodo Dragon Populations
Source: PLoS One. 2012 Sep 19;7(9):e45398. doi: 10.1371/journal.pone.0045398 (PMC3446886; doi:10.1371/journal.pone.0045398)
Supplement: Methods S2 — Methods & Results. (DOCX) [file pone.0045398.s004.docx]

**Supplementary Methods & Results S2:**

*Covariate effects on growth*

Methods: A GAMM was used to assess the relative effects of all available covariates on growth rate. Covariates modelled were sex, site, year, mean size (snout-vent-length; cm SVL), and recapture interval (years). The year covariate represents calendar year (including midpoints) for the growth rate estimate and accounts for the implicit time-dependency in the sampling design. Recapture interval was removed from the final analysis as it did not significantly strengthen the fit of the overall model.

Results: The GAMM assessing spatial and temporal variation in growth included annual growth rate as the response variable along with four possible predictors of growth: mean body size, mean growth year, sex, and site (Table 1, Figure 3, Figure S1, Figure S2). The model was generally a good fit to the data accounting for 36.4% of the variance in growth rate. The analysis suggests dragon growth rates are dependent on all covariates (Table 1), although age and cohort effects cannot be separated within the year effect. Remaining unexplained variability suggests factors other than those investigated, such as population genetics, may also be affecting growth rates.

Temporal variability in growth

The year effect (Table 1) suggests a significant inter-annual variability in growth, which would infer a varying environmental influence between years. The GAMM plot for year effect however (Figure S1), shows a fluctuating wave-like pattern with slightly lower growth rates prior to 2004 and around 2010. Considering the very small range of growth rate deviation against this predictor it is unlikely to be a highly significant influence on growth rate in this species.
