# Supplementary material for: Life-History and Spatial Determinants of Somatic Growth Dynamics in Komodo Dragon Populations
Source: PLoS One. 2012 Sep 19;7(9):e45398. doi: 10.1371/journal.pone.0045398 (PMC3446886; doi:10.1371/journal.pone.0045398)
Supplement: Table S1 — (DOCX) [file pone.0045398.s005.docx]

Table S1. Demographic summary of the Komodo dragons from which growth records were obtained.

| **Island** | **Komodo** | | | | **Rinca** | | | | **Gili Motang** | **Nusa Kode** | |  |
| --- | --- | --- | --- | --- | --- | --- | --- | --- | --- | --- | --- | --- |
| **Site** | Lla | Lli | Lse | Lwa | Lba | Lbu | Lda | Lto | Gm | Nk | **Total** | |
| **Records** | 101 | 125 | 76 | 47 | 105 | 176 | 80 | 92 | 31 | 6 | 839 | |
| **Dragons** | 56 | 58 | 36 | 19 | 50 | 77 | 35 | 47 | 17 | 5 | 400 | |
| **Males** | 23 | 35 | 12 | 9 | 34 | 38 | 17 | 26 | 4 | 3 | 201 | |
| **Females** | 16 | 11 | 9 | 2 | 4 | 16 | 4 | 8 | 7 | 0 | 77 | |

*Notes:* Provided are the number of growth records, individuals sampled, known males and known females that constitute all growth records for each site.
