# Supplementary material for: Life-History and Spatial Determinants of Somatic Growth Dynamics in Komodo Dragon Populations
Source: PLoS One. 2012 Sep 19;7(9):e45398. doi: 10.1371/journal.pone.0045398 (PMC3446886; doi:10.1371/journal.pone.0045398)
Supplement: Table S2 — (DOCX) [file pone.0045398.s006.docx]

Table S2. Site covariates for modelling spatial variation in growth rate of Komodo dragons.

|  |  | **Covariates** |  |
| --- | --- | --- | --- |
| **Site** | Population density | Ungulate prey availability | Inbreeding Coefficient |
| Gili Motang (Gm) | 14.29 | 5.60 | 0.26 |
| Loh Baru (Lba) | 27.12 | 23.58 | 0.02 |
| Loh Buaya (Lbu) | 31.42 | 15.24 | 0.15 |
| Loh Dasami (Lda) | 20.80 | 20.52 | 0.09 |
| Loh Lawi (Lla) | 18.87 | 20.42 | 0.01 |
| Loh Liang (Lli) | 40.08 | 30.71 | 0.12 |
| Loh Sebita (Lse) | 24.85 | 23.77 | -0.07 |
| Loh Tongker (Lto) | 37.69 | 25.38 | -0.10 |
| Loh Wau (Lwa) | 41.75 | 34.55 | -0.07 |
| Nusa Kode (Nk) | 16.01 | 7.81 | -0.34 |
